# Challenges of using the Occupational Asthma-specific job exposure matrix to assess occupational exposure and hand eczema

**DOI:** 10.3389/falgy.2026.1838194

**Published:** 2026-07-21

**Authors:** Marjolein J. Brands, Laura Loman, Ute Bültmann, Marie L. A. Schuttelaar

**Affiliations:** 1 Department of Dermatology, University of Groningen, University Medical Center Groningen, Groningen, Netherlands; 2 Department of Health Sciences, Community and Occupational Medicine, University of Groningen, University Medical Center Groningen, Groningen, Netherlands

**Keywords:** allergens, contact dermatitis, hand dermatitis, irritants, job exposure index, job exposure matrix, occupational hand eczema, sensitizers

## Abstract

**Background:**

General population-based studies addressing the association between occupational exposure to irritants and allergens and hand eczema (HE) are limited, or only focus on specific occupations using self-reported exposure data.

**Objectives:**

To assess the association between occupational exposure to irritants and allergens and moderate-to-very severe HE, by using the Occupational Asthma-specific job exposure matrix (OasJEM) within the Dutch general population, and to address the applicability of this approach.

**Methods:**

Within the Lifelines Cohort Study, participants with moderate-to-very severe HE at worst in the past year vs. no HE in lifetime were identified based on self-reports. The OasJEM was used to link occupations with occupational exposure to irritants and sensitizers, relying on expert-based classification.

**Results:**

In total, 56,978 (41.9%) participants were included. The multivariate binary logistic regression analyses showed associations between occupational exposure to irritants [Odds ratio (OR): 1.19 (95% CI: 1.06–1.33)], high molecular weight sensitizers [OR: 1.20 (95% CI: 1.06–1.36)], mites [OR: 1.41 (95% CI: 1.16–1.73)] and disinfectants and cleaning products [1.25 (95% CI: 1.11–1.42)], and moderate-to-very severe HE.

**Conclusions:**

While associations between several irritants and allergens and HE were found by applying the OasJEM, which may provide insights into occupational exposure patterns and highlight the general relevance of occupational exposure in relation to HE and its prevention, several methodological and conceptual challenges must be acknowledged. A JEM not specifically designed for HE, like the OasJEM, may not fully capture the complex interplay between occupational exposure and HE, particularly in reflecting direct skin contact, the primary exposure route relevant for HE. However, in the absence of widely available and reliable HE-specific exposure assessment tools, the use of non-HE-specific JEMs may be considered a pragmatic alternative, while acknowledging certain limitations. Future studies should focus on task-based and HE-specific exposure data, providing more accurate insights into occupational exposure and HE.

## 1 Introduction

Hand eczema (HE) is a common skin disease, both in the general and in the working population. In the European general population, HE has a lifetime prevalence of 14.5% (1), with up to 60% of cases considered to be work-related (2). The pathogenesis of HE is multifactorial, involving endogenous and exogenous risk factors (3). The most relevant exogenous risk factor for HE includes exposure to irritants and allergens, which predominantly occurs in occupational settings (3). Repeated or prolonged exposure to irritants, such as wet work, soaps or disinfectants, may lead to irritant contact dermatitis (ICD) of the hands, while exposure to allergens, such as fragrances, acrylates or epoxy resins, may lead to allergic contact dermatitis (ACD) of the hands in sensitized individuals, mediated by a type IV hypersensitivity (4, 5). Additionally, exposure to type I allergens may cause IgE mediated sensitization, resulting in contact urticaria, or with ongoing exposure, protein contact dermatitis of the hands (3).

Research on occupational exposure and HE often relies on retrospective self-reported data (4, 5). Another approach is using a job exposure matrix (JEM), which links job titles to exposure estimates, minimizing reporting, recall and differential bias (6). Recently, a JEM has been used to assess the association between wet work HE. However, wet work represents only one of several relevant causes of irritant contact dermatitis, and no JEMs currently address other irritant exposures, or exposure to allergens (7–9). For asthma, multiple JEMs are available, including the Occupational Asthma-specific JEM (OasJEM), which incorporates expert-based information on occupational exposure to 30 irritants and sensitizers (Table 1) (10, 11). Although the OasJEM was developed to assess exposure to irritants and sensitizers associated with occupational asthma, its application may be extended to occupational HE, given the substantial overlap between agents that act as respiratory allergens or irritants and those known to elicit or exacerbate HE. For example, irritant substances such as bleach, ammonia or organic solvents, can cause both ICD and irritant-induced occupational asthma, while substances such as persulfates or epoxy resins are well-recognized occupational sensitizers that may induce both ACD and occupational allergic asthma (4, 5, 10, 11). In addition, enzymes or food proteins may induce contact urticaria and protein contact dermatitis of the hands, while inhalation may induce respiratory IgE-mediated sensitization and occupational allergic asthma (10–12). Together, these examples illustrate that the application of the OasJEM may be extended to occupational HE. However, to date, the OAsJEM has not been used to study the association between occupational exposure and HE in the general population. Therefore, the aim of the present study was to assess the association between occupational exposure to irritants and allergens and moderate-to-very severe HE within the Dutch general population, by applying the OasJEM, and to address the applicability of this approach.

**Table 1:** Categorization of individual occupational exposure agents from the Occupational Asthma-specific job exposure matrix (OasJEM), with examples of associated occupations.^a^

Group	Individual agents	Examples of occupations with exposure	References
Irritants	Textiles, moulds, endotoxin, high-level chemical disinfectants, aliphatic amines, isocyanates, acrylates, epoxy resins, persulfates/henna, wood, metal, metal working fluids, herbicides, insecticides, fungicides, indoor cleaning, bleach, organic solvents, exhaust fumes	Hairdressers, butchers, varnishers	Le Moual et al. (10)
HMW sensitizers	Animals, fish/shellfish, flour, foods, plants-related dusts, house dust mites, storage mites, plant mites, enzymes, latex, textiles, drugs, wood	Domestic helpers and cleaners, bakers, cooks	Le Moual et al. (10)
LMW sensitizers	Drugs, high-level chemical disinfectants, aliphatic amines, isocyanates, acrylates, epoxy resins, persulfates/henna, wood, metal, metal working fluids	Pharmaceutical assistants, plumbers and pipe fitters, motor vehicle mechanics	Le Moual et al. (10)
Mites	House dust mites, storage mites, plant mites	Housekeepers, gardeners, agricultural labourers	Le Moual et al. (10)
Microbial exposure	Textiles, moulds, endotoxin, metal working fluids	Garbage collectors, dairy-products makers, machine tool operators	Le Moual et al. (10)
Highly reactive chemicals	High-level chemical disinfectants, aliphatic amines, isocyanates, acrylates, epoxy resins, persulfates/henna, bleach, organic solvents	Nursing and midwifery associate professionals, beauticians, painters	Le Moual et al. (10)
Biocides	High-level chemical disinfectants, herbicides, insecticides, fungicides, bleach	Agricultural labourers, wood treaters, gardeners	Le Moual et al. (10)
Disinfectants and cleaning products	Indoor cleaning, bleach, high-level chemical disinfectants	Nurses, domestic helpers and cleaners, personal care workers	Sit et al. (20)

HMW, high molecular weight; LMW, low molecular weight.

a Categorization of occupational exposures from the Occupational Asthma-specific Job exposure matrix (OasJEM) (10, 11).

## 2 Materials and methods

### 2.1 Design and population

This cross-sectional add-on study was conducted within the Lifelines Cohort Study (13). The design of the present study has been described in more detail previously (9, 14). In brief, a digital, self-administered questionnaire on skin diseases, including HE, was distributed to 135,950 adult participants in 2020. The response rate for the question on the lifetime prevalence of HE was 42.5% (*n* = 57,796) (14). In the present study, only participants with self-reported moderate-to-very severe HE at worst in the past year, as assessed using a validated question and photographic guide, or with no HE in lifetime, that provided a description of their occupation between 2006 (the initial year of the Lifelines Cohort Study) and 2022, were included (9, 15, 16). By focusing exclusively on participants with at least moderate HE, we aimed to reduce potential inaccuracies associated with self-reports of HE, as individuals with more severe symptoms are generally more likely to recognise and accurately report their condition (17). Given this large population-based cohort and the use of self-reported data, obtaining reliable information on etiological subtypes of HE was not feasible. Therefore, no stratification by etiological subtype was obtained. Details on the questions and categorizations of the variables used in the present study can be found in Supplementary Table S1 and in previously published work (9, 14). Data collection was conducted according to the guidelines of the Declaration of Helsinki. All procedures were approved by the Medical Ethics Committee of the University Medical Center Groningen (reference number: METc 2007/152, reference number current add-on study: METc 2019/571).

### 2.2 The Occupational Asthma-specific job exposure matrix

Occupational exposure to irritants and allergens was assigned by using a JEM, a tool which links job titles to exposure estimates. In the present study, the OasJEM was used, which was originally developed in 2000, and updated in 2018 (10, 11). For each occupation classified according to the International Standard Classification of Occupations, version 1988 (ISCO-88), the JEM includes data on the level of occupational exposure to agents that are known to be associated with occupational asthma (18). The selection of irritants and allergens within this JEM was based on available literature and previously developed JEM's. The JEM includes information on a total of 30 individual agents, which were additionally categorised into seven occupational exposure groups known to be associated with occupational asthma [irritants, high molecular weight (HMW) sensitizers, low molecular weight (LMW) sensitizers, mites, microbial exposure, highly reactive chemicals and biocides]. This grouping was initially based on previous work by Chan-Yeung et al., which stratified over 150 chemical and biological agents into high and low molecular weight categories, and was further supplemented by recent literature and expert consensus (10, 11, 19). Based on previous literature also using the OasJEM, one additional group was defined within the present study (disinfectant and cleaning products) (20). For each agent included in the OAsJEM (e.g., high-level chemical disinfectants, aliphatic amines, isocyanates or acrylates), exposure assignments for occupations classified according to ISCO-88 were evaluated by working groups consisting of three out of eight experts. Occupational exposure was classified into three categories: “no exposure”, “medium level of exposure” (low-to-moderate probability or low intensity) or “high level of exposure” (high probability of exposure with moderate-to-high intensity), based on expert classification. Experts initially assessed exposures independently, after which inconsistencies were resolved through consensus meetings. In constructing the JEM, specificity was prioritized over sensitivity, which means that occupations were classified as highly exposed only when both the probability and intensity of exposure were considered sufficiently high. A final review by all experts was subsequently performed to evaluate assigned exposures and refine recommendations if necessary. In the present study, the most recently self-reported occupation, classified according to the International Standard Classification of Occupations, version 2008 (ISCO-08), was extracted from the Lifelines baseline and follow-up questionnaires (2006–2022), and occupational exposure was assigned by linking data from the OasJEM to these ISCO-08 codes (18). Details on the individual agents and exposure groups used in the present study can be found in Table 1.

### 2.3 Statistical analyses

The association between occupational exposure and moderate-to-very severe HE at worst in the past year versus participants with no lifetime history of HE were examined with univariate and multivariate binary logistic regression analyses, adjusted for sex, age and atopic dermatitis (21, 22). The analyses were performed for all individual agents from the OAsJEM, as well as for the grouped categorized agents presented in Table 1. Results were expressed in odds ratios (ORs) with 95% confidence intervals (95% CIs). Binary logistic regression analyses were only performed when at least 100 cases and controls were available. A *p*-value of <0.05 was deemed statistically significant. Each occupational exposure group and individual agent was analysed as medium and high exposure versus no exposure, and as dichotomized into exposure versus no exposure categories. Data analyses were performed using the Statistical Products and Service Solutions package version 28 (SPSS Inc., Chicago, IL, U.S.A.).

## 3 Results

### 3.1 Population characteristics

In total, 56,978 (41.9%) participants were included. The self-reported one-year prevalence of HE was 7.3% (*n* = 4 170), and the self-reported lifetime prevalence of HE was 15.0% (*n* = 8,555). Moderate-to-very severe HE at worst in the past year was reported by 1,572 (37.7%) of those participants who reported HE in the past year. Self-reported physician-diagnosed atopic dermatitis was reported in 5,145 (9.2%) of the total study population, in 710 (45.2%) of participants with moderate-to-very severe hand eczema in the past year, and in 2 724 (5.7%) of participants with no HE in lifetime. ISCO-08 classified occupational groups, along with the predefined confounders (sex, age and atopic dermatitis) and occupational exposure according to the OasJEM, showed significant differences between participants with moderate-to-very severe HE in the past year and those who reported no HE in lifetime (Tables 2, 3).

**Table 2:** Characteristics of participants; total study population and stratified by self-reported moderate-to-very severe hand eczema at worst in the past year vs. no hand eczema in lifetime (*N* = 56,978).

	Total, *n* (%) (*N* = 56,978)	Moderate-to-very severe HE past year, *n* (%) (*N* = 1,572)	No HE ever, *n* (%) (*N* = 48,423)	*p*-value
Sex				
Female	34,348 (60.3)	1,146 (72.9)	28,223 (58.3)	**<**.**001**
Male	22,630 (39.7)	426 (27.1)	20,200 (41.7)	
Missing	0	0	0	
Age in years, mean ± SD	55.5 ± 12.4	49.2 ± 12.0	55.9 ± 12.5	**<**.**001**
Missing	0	0	0	
Year of most recently reported ISCO-08 occupation				
2006–2011	900 (1.6)	27 (1.7)	772 (1.6)	.70
2011–2016	24,710 (43.4)	648 (41.2)	21,248 (43.9)	.**04**
2016–2021	28,296 (49.7)	803 (51.1)	23,833 (49.2)	.15
2021–2023	3,041 (5.3)	94 (6.0)	2,544 (5.3)	.21
Missing	31	0	26	
ISCO-08 major occupation groups				
Managers	3,633 (6.4)	69 (4.4)	3,198 (6.6)	**<**.**001**
Professionals	15,337 (26.9)	397 (25.3)	13,012 (26.9)	.15
Technicians and associate professionals	10,756 (18.9)	314 (20.0)	9,039 (18.7)	.19
Clerical support workers	7,030 (12.3)	198 (12.6)	6,022 (12.4)	.85
Service and sales workers	10,940 (19.2)	372 (23.7)	9,086 (18.8)	**<.001**
Skilled agricultural, forestry and fishery workers	1,243 (2.2)	29 (1.8)	1,087 (2.2)	.29
Craft and related trades workers	3,271 (5.7)	57 (3.6)	2,894 (6.0)	**<**.**001**
Plants and machine operator and assemblers	1,821 (3.2)	34 (2.2)	1,631 (3.4)	.**01**
Elementary occupations	2,858 (5.0)	100 (6.4)	2,374 (4.9)	.**01**
Armed forces occupations	89 (0.2)	<10 (<0.6)	80 (0.2)	1.00
Missing	0	0	0	
Atopic dermatitis^a^	5,145 (9.2)	710 (45.2)	2,724 (5.7)	**<**.**001**
Missing	1,001	80	583	

HE, hand eczema; ISCO-08, International Standard Classification of Occupations version 2008; n, number; SD, Standard deviation.

*p*-values ≤0.05 are shown in bold.

a Self-reported physician diagnosed atopic dermatitis.

**Table 3:** Occupational exposure according to the Occupational Asthma-specific job exposure matrix (groups and individual occupational exposure agents); total study population and stratified by self-reported moderate-to-very severe hand eczema at worst in the past year vs. no hand eczema in lifetime (*N* = 56,978).

	Total, *n* (%) (*N* = 56,978)	Moderate-to-very severe HE past year, *n* (%) (*N* = 1,572)	No HE ever, *n* (%) (*N* = 48,423)	*p*-value
Occupational exposure (groups)^a^				
Irritants	21,017 (37.1)	644 (41.2)	17,603 (36.6)	**<.001**
HMW sensitizers	12,476 (22.0)	409 (26.1)	10,273 (21.4)	**<.001**
LMW sensitizers	12,278 (21.7)	382 (24.4)	10,215 (21.2)	.**003**
Mites	4,012 (7.1)	124 (7.9)	3,331 (6.9)	.13
Microbial exposure	2,175 (3.8)	49 (3.1)	1,904 (4.0)	.10
Highly reactive chemicals	14,027 (24.8)	439 (28.1)	11,638 (24.2)	**<**.**001**
Biocides	10,720 (18.9)	357 (22.8)	8,871 (18.4)	**<**.**001**
Disinfectants and cleaning products	13,704 (24.2)	478 (30.5)	11,217 (23.3)	**<**.**001**
Occupational exposure (individual agents)^a^				
Animals	1,148 (2.0)	31 (2.0)	988 (2.1)	.84
Fish/shellfish	425 (0.8)	12 (0.8)	368 (0.8)	.99
Flour	309 (0.5)	<10 (<0.6)	268 (0.6)	.56
Foods	326 (0.6)	<10 (<0.6)	284 (0.6)	.47
Plants-related dusts	703 (1.2)	13 (0.8)	625 (1.3)	.11
House dust mites	2,362 (4.2)	84 (5.4)	1,894 (3.9)	.**004**
Storage mites	1,169 (2.1)	30 (1.9)	1,006 (2.1)	.63
Plant mites	703 (1.2)	13 (0.8)	625 (1.3)	.11
Enzymes	309 (0.5)	<10 (<0.6)	268 (0.6)	.56
Latex	8,542 (15.1)	312 (19.9)	6,899 (14.3)	**<**.**001**
Textiles	223 (0.4)	<10 (<0.6)	194 (0.4)	.61
Moulds	1,587 (2.8)	35 (2.2)	1,389 (2.9)	.13
Endotoxin	1,647 (2.9)	38 (2.4)	1,443 (3.0)	.19
Drugs	536 (0.9)	17 (1.1)	449 (0.9)	.54
High-level chemical disinfectants	8,385 (14.8)	293 (18.7)	6,874 (14.3)	**<**.**001**
Aliphatic amines	970 (1.7)	33 (2.1)	799 (1.7)	.18
Isocyanates	378 (0.7)	<10 (<0.6)	348 (0.7)	.12
Acrylates	641 (1.1)	14 (0.9)	563 (1.2)	.32
Epoxy resins	630 (1.1)	10 (0.6)	568 (1.2)	.**049**
Persulfates/henna	587 (1.0)	25 (1.6)	458 (1.0)	.**01**
Wood	685 (1.2)	11 (0.7)	607 (1.3)	.**05**
Metal	1,919 (3.4)	34 (2.2)	1,677 (3.5)	.**005**
Metal working fluids	256 (0.5)	<10 (<0.6)	226 (0.5)	.39
Herbicides	734 (1.3)	13 (0.8)	659 (1.4)	.07
Insecticides	1,840 (3.2)	48 (3.1)	1,612 (3.4)	.54
Fungicides	1,434 (2.5)	33 (2.1)	1,251 (2.6)	.23
Indoor cleaning	10,985 (19.4)	397 (25.4)	8,942 (18.6)	**<**.**001**
Bleach	6,963 (12.3)	251 (16.0)	5,669 (11.8)	**<**.**001**
Organic solvents	6,333 (11.2)	179 (11.4)	5,319 (11.1)	.64
Exhaust fumes	3,778 (6.7)	85 (5.4)	3,355 (7.0)	.**02**
Number of exposures to individual agents, mean ± SD	1.2 ± 1.9	1.3 ± 1.9	1.1 ± 1.9	**<**.**001**
Missing	357	<10	327	

HE, hand eczema; HMW, high molecular weight; LMW, low molecular weight; n, number; SD, standard deviation.

*P*-values ≤0.05 are shown in bold.

a Characteristics derived from linkage of the Occupational Asthma-specific job exposure matrix by Kennedy et al. and Le Moual et al., according to most recently reported International Standard Classification of Occupations version 2008 (ISCO-08) coded occupation (10, 11, 18). A correspondence table provided by the International Labour Organization was used to align the ISCO-08 codes with the ISCO 1988 (ISCO-88) (18). In cases where ISCO-08 codes could be converted to multiple ISCO-88 codes, the ISCO-88 with the most suitable description for the occupation was selected.

### 3.2 Associations between occupational exposure and moderate-to-very severe hand eczema

#### 3.2.1 Occupational exposure groups

**Figure 1:**
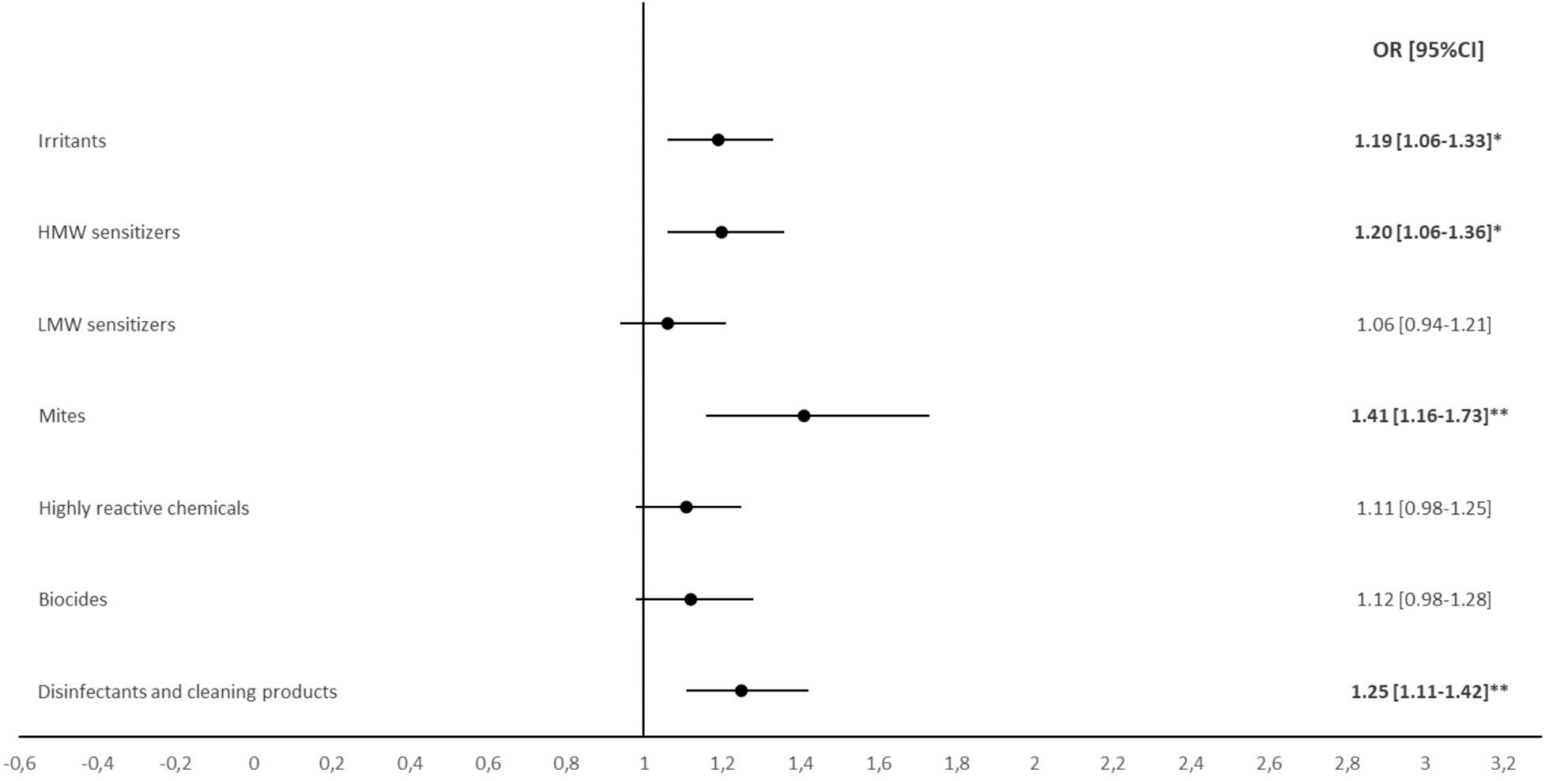
Associations between occupational exposure (groups) and self-reported moderate-to-very severe hand eczema at worst in the past year vs. no self-reported hand eczema in lifetime (*N* = 49 995). Adjusted for sex, age and self-reported physician diagnosed atopic dermatitis. Data on occupational exposure was derived from linkage with the Occupational Asthma-specific job exposure matrix by Kennedy et al. and Le Moual et al., according to most recently reported International Standard Classification of Occupations version 2008 (ISCO-08) coded occupation (10, 11, 18). The categorization of occupational exposure agents is presented in Table 1. Binary logistic regression analyses were only performed when at least 100 cases and controls were available. *p*-values ≤0.05 are shown in bold. *Significant at 0.05 level, **Significant at 0.001 level. CI, confidence interval; HMW, high molecular weight; LMW, low molecular weight; OR, odds ratio.

Occupational exposure to irritants [adjusted OR: 1.19 (95% CI: 1.06–1.33)], HMW sensitizers [adjusted OR: 1.20 (95% CI: 1.06–1.36)], mites [adjusted OR: 1.41 (95% CI: 1.16–1.73)] and disinfectants and cleaning products [adjusted OR: 1.25 (95% CI: 1.11–1.42)] were all associated with moderate-to-very severe HE in the past year, while no associations were found for LMW sensitizers, highly reactive chemicals and biocides (Figure 1 and Supplementary Table S2).

Both medium and high levels of occupational exposure to irritants were associated to moderate-to-very severe HE [adjusted OR: 1.17 (95% CI: 1.02–1.33) and adjusted OR: 1.22 (95% CI: 1.05–1.43), respectively] (Supplementary Table S3). Similarly, exposure to the group disinfectants and cleaning products showed significant associations [adjusted OR: 1.22 (95% CI: 1.06–1.41) and adjusted OR: 1.32 (95% CI: 1.10–1.59), respectively]. For highly reactive chemicals and biocides, only high exposure was associated [adjusted OR: 1.26 (95% CI: 1.05–1.50) and adjusted OR: 1.28 (95% CI: 1.06–1.55), respectively]. Medium, but not high exposure to HMW sensitizers was associated with moderate-to-very severe HE [adjusted OR: 1.22 (95% CI: 1.05–1.43)].

#### 3.2.2 Individual occupational exposure agents

**Figure 2:**
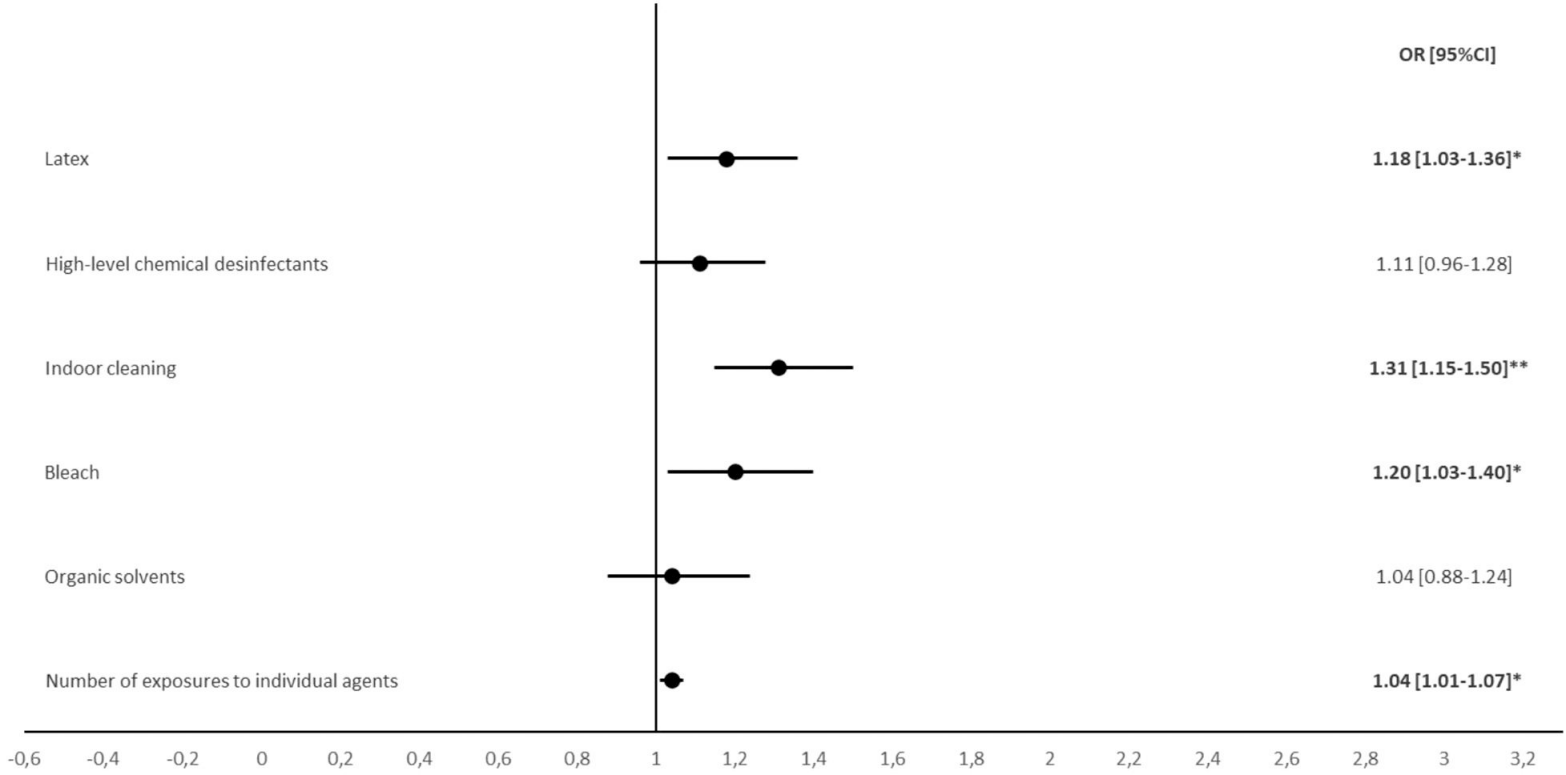
Associations between occupational exposure (individual agents) and self-reported moderate-to-very severe hand eczema at worst in the past year vs. no self-reported hand eczema in lifetime (*N* = 49,995). Adjusted for sex, age and self-reported physician diagnosed atopic dermatitis. Data on occupational exposure was derived from linkage with the Occupational Asthma-specific job exposure matrix by Kennedy et al. and Le Moual et al., according to most recently reported International Standard Classification of Occupations version 2008 (ISCO-08) coded occupation (10, 11, 18). Binary logistic regression analyses were only presented when at least 100 cases and controls were available. *P*-values ≤0.05 are shown in bold. *Significant at 0.05 level, **Significant at 0.001 level. CI, confidence interval; OR, Odds ratio.

Occupational exposure to several individual agents was associated with moderate-to-very severe HE in the past year [adjusted OR: 1.04 (95% CI: 1.01–1.07)] (Figure 2 and Supplementary Table S2). In addition, exposure to latex, indoor cleaning products and bleach were associated with moderate-to-very severe HE [adjusted OR: 1.18 (95% CI: 1.03–1.36), adjusted OR: 1.31 (95% CI: 1.15–1.50) and adjusted OR: 1.20 (95% CI: 1.03–1.40), respectively]. No associations were found for high-level chemical disinfectants and organic solvents.

Both medium and high occupational exposure to indoor cleaning products were associated with moderate-to-very severe HE [adjusted OR: 1.33 (95% CI: 1.14–1.55) and adjusted OR: 1.29 (95% CI: 1.05–1.57), respectively] (Supplementary Table S3). High level of exposure to bleach was associated with moderate-to-very severe HE [adjusted OR: 1.27 (95% CI: 1.02–1.58)], while no association was found for medium exposure.

## 4 Discussion

The present study assessed associations between occupational exposure and HE by applying the OasJEM, and identified several associations with irritants, HMW sensitizers, mites and disinfectants and cleaning products.

The observed associations for irritants and disinfectants/cleaning products correspond with what is known about their role in the pathogenesis of HE. The exposure group of irritants includes individual exposure agents that could, in relation to HE, be either primarily irritant, such as bleach, or both irritant and contact allergenic through direct skin contact, e.g., high level chemical disinfectants such as chlorhexidine gluconate, and may contribute to the development of ICD and/or ACD of the hands (Table 1) (4, 5, 10, 23). Individual agents within the disinfectants/cleaning products group (e.g., indoor cleaning products such as ammonia) primarily act as irritants upon direct skin contact, but may also act as contact allergens, for example due to added fragrances or preservatives (4, 5, 23). In contrast, associations with LMW sensitizers, highly reactive chemicals and biocides were also anticipated, as these groups contain agents such as organic solvents (e.g., acetone) who are primarily irritant, and metal working fluids, and isocyanates, which may act as irritants and contact allergens (3–5). However, no such association was observed in this study. Furthermore, the exposure group of HMW sensitizers and mites mainly include several type 1 allergens, such as house dust mites, which have been reported to elicit eczematous skin reactions on nonlesional skin in patients with AD upon epicutaneous application (within an experimental and prospective multicenter trial) (24, 25). The observed association between HMW sensitizers and mites and HE may, in addition, be explained by the role of individual agents from these specific exposure groups, such as type I allergens like fish/shellfish, flour, foods or latex (Table 1), in the pathogenesis of contact urticaria/protein contact dermatitis (3).

Assessment of occupational exposure and the application of the OasJEM for HE comes with a few methodological and conceptual challenges. First, while a JEM standardizes occupational exposure assessment, the OasJEM may not fully capture HE-specific exposure pathways, as it was designed for occupational asthma, and does not necessarily involve exposure via skin contact. This could have led to exposure misclassification, potentially affecting the strength and clarity of the observed association between occupational exposure and HE. Second, occupational exposure to irritants and allergens is inherently complex, with various factors potentially interacting in its association with HE. For instance, exposure to irritants may contribute to impairment of the skin barrier, which has been suggested to increase the risk of ACD upon exposure to allergens (4, 26, 27). In this context, it would also be valuable to have a combined JEM that, in addition to the exposures considered in this study, also provides information on wet work, for which only a separate, specific JEM currently exists (7). However, similar to HE, both irritant and allergic mechanisms may also coexist in occupational asthma, as agents (e.g., isocyanates) have the potential to act as both airway irritant and sensitizer, and irritant exposure may even facilitate subsequent allergic sensitization (4, 5, 10, 12, 28). Third, assessing the contribution of individual agents is challenging due to co-exposure (e.g., simultaneous exposure to multiple irritants and allergens in professions such as hairdressing or metalworking), and the observed associations may not reflect a direct causal relationship (e.g., exposure to irritants or allergens may prompt heightened hygiene measures and glove use, possibly also explaining the association between mite and HMW sensitizers and HE). This, however, also applies to the use of the OasJEM in the context of occupational asthma, where the observed associations do not necessarily indicate a causal relationship. Nonetheless, the findings of the present study highlight the importance of a thorough occupational history by dermatologists and/or occupational physicians in patients with HE, taking into account a wide range of occupational irritants and contact allergens, including those traditionally associated with occupational asthma. By expanding the focus to include a detailed occupational history, preventive measures may be more effectively tailored to the patient's unique work environment.

Considering the previously outlined methodological and conceptual challenges, other studies assessing occupational exposure and its association with different types of eczema using the OasJEM have reported findings that are inconsistent with those from the present study. For example, one study focusing on Canadian workers who filed claims due to occupational contact dermatitis found, in addition to associations with individual agents from the irritants group, associations with several agents from the LMW sensitizers and the highly reactive chemicals group (e.g., aliphatic amines, metal, metal working fluids, persulfates/henna and organic solvents) (29). Another Australian population-based study only found an association between microbial exposure and non-atopic eczema, with no associations observed for other exposure groups (30). These inconsistencies with the present study may reflect differences in study populations or design [e.g., the diagnosis of HE, contact dermatitis, or (non-)atopic dermatitis based on self-reports vs. work claims]. However, these inconsistencies may also result from using a JEM not specifically designed for skin diseases, as noted earlier.

This study has several strengths, including the comprehensive assessment of a broad range of occupational exposures and inclusion of a large general population sample. While a JEM could reduce recall bias, several limitations should also be acknowledged, in addition to the broader conceptual and methodological challenges previously discussed. First, a JEM may not completely capture variations in exposure among individuals within the same occupation. The precision of the OasJEM could be enhanced by implementing the expert assessment step recommended; however, this was not feasible due to the large sample size (10). In addition, it was not possible to distinguish between clinically verified diagnoses of ICD and ACD of the hands, and detailed information on potential contact allergens relevant to participants' HE (including participants' knowledge of personally relevant contact allergies), and their treatment history was lacking. At last, although the Lifelines cohort has been shown to be broadly representative of the population in Northern Netherlands, the response rate of 42.5% may have resulted in selection bias, representing a potential limitation of the study (31).

In conclusion, the findings of this study in general highlight the relevance of occupational exposure in relation to HE and its prevention. However, while the OasJEM may provide general insights into occupational exposure patterns, this study highlights key methodological and conceptual challenges in capturing the complex interplay between occupational exposure and HE by using the OasJEM, particularly in reflecting direct skin contact, the primary exposure route for HE. However, in the absence of widely available and reliable HE-specific exposure assessment tools, the use of non-HE-specific JEMs may be considered a pragmatic alternative, while acknowledging certain limitations. Future studies should focus on task-based and HE-specific exposure data, providing more accurate insights into the association between occupational exposure to irritants and allergens and HE, ultimately aiming to facilitate the development of targeted prevention strategies to reduce the burden of HE.

## Data Availability

The datasets presented in this article are not readily available because data may be obtained from a third party and are not publicly available. Researchers can apply to use the Lifelines data used in this study. More information about how to request Lifelines data and the conditions of use can be found on their website (https://www.lifelines-biobank.com/researchers/working-with-us/step-1-prepare-and-submit-your-application).
