# Supplementary material for: Challenges of using the Occupational Asthma-specific job exposure matrix to assess occupational exposure and hand eczema
Source: Front Allergy. 2026 Jul 21;7:1838194. doi: 10.3389/falgy.2026.1838194 (PMC13433430; doi:10.3389/falgy.2026.1838194)
Supplement: Supplementary file 1 [file Table1.docx]

Challenges of Using the Occupational Asthma-specific Job Exposure Matrix to Assess Occupational Exposure and Hand Eczema

**Marjolein J. Brands^1^, Laura Loman^1^, Ute Bültmann^2^, Marie L.A. Schuttelaar^1*^**

^1^Department of Dermatology, University of Groningen, University Medical Center Groningen, Groningen, The Netherlands.

^2^Department of Health Sciences, Community and Occupational Medicine, University of Groningen, University Medical Center Groningen, The Netherlands.

# *Correspondence to: Prof. Dr. Marie L. A. Schuttelaar, Department of Dermatology, University of Groningen, University Medical Center Groningen, Groningen, The Netherlands. E-mail: [m.l.a.schuttelaar@umcg.nl](mailto:m.l.a.schuttelaar@umcg.nl).

## Supplementary Tables

**Table S1.** Questions and response options including references.

**Table S2.** Associations between occupational exposure and moderate-to-very severe hand eczema at worst in the past year vs no hand eczema in lifetime (N=49 995).

**Table S3.** Associations between different levels of occupational exposure and moderate-to-very severe hand eczema at worst in the past year vs no hand eczema in lifetime (N=49 995).

**Table S1.** Questions and response options including references.^a^

| **Topic** | **Question and response options (English translation)** | **References** |
| --- | --- | --- |
| Sex | What is your sex?   - Male (1) - Female (2) | None. |
| Age | What is your year of birth? | None. |
| Lifetime prevalence of hand eczema | Hand eczema is a skin disease of the hands. Symptoms are redness, desquamation/dry skin and sometimes vesicles and/or fissures. HE can cause itch and/or pain.  Have you ever (now or in the past) had hand eczema?   - Yes (1) - No (2) | Nordic Occupational Skin Questionnaire (NOSQ)-2002; question D1, modified.^1^ |
| 1-year prevalence of hand eczema | Have you had hand eczema in the past 12 months?   - Yes (1) - No (2) | Meding et al. 2010 and Smit et al. 1992^2,3^ |
| Severity of hand eczema | The adapted photographic guide, containing 16 photographs distributed in 4 groups of severity of hand eczema was used to assess self-reported severity of hand eczema.  Which group did your HE match, when your hand eczema was at its worst in the past 12 months?   - Almost clear (1) - Moderate (2) - Severe (3) - Very severe (4) | Coenraads et al.^4^; Hald et al.^5^ |
| Most recently reported International standard classification of occupations version 2008 (ISCO-08) classified occupation | If you have (had) a paid job, what is your last or current profession?  What is your current profession?  Answers were coded according to the ISCO-08 classification | International Standard Classification of Occupations 2008^6^ |
| Atopic dermatitis | Have you ever been diagnosed with atopic dermatitis or atopic eczema by a doctor?   - Yes (1) - No (2) | Barbarot et al. 2018; question P1.^7^ |

^a^Supplementary Table S1 was previously published in adapted form.^8-14^

**Table S2.** Associations between occupational exposure and moderate-to-very severe hand eczema at worst in the past year vs no hand eczema in lifetime (N=49 995).

|  | **Moderate-to-very severe HE in the past year, n (%) (n=1572)** | **No HE ever, n (%) (n=48423)** | **Crude OR (95% CI)** | ***P-*value** | **Adjusted OR^†^ (95% CI)** | ***P-*value** |
| --- | --- | --- | --- | --- | --- | --- |
| **Occupational exposure (groups)** | | | | | | |
| Irritants  HMW sensitizers  LMW sensitizers  Mites  Microbial exposure  Highly reactive chemicals  Biocides  Disinfectants and cleaning products | 644 (41.2)  409 (26.1)  382 (24.4)  124 (7.9)  49 (3.1)  439 (28.1)  357 (22.8)  478 (30.5) | 17603 (36.6)  10273 (21.4)  10215 (21.2)  3331 (6.9)  1904 (4.0)  11638 (24.2)  8871 (18.4)  11217 (23.3) | **1.21 (1.09-1.34)**  **1.30 (1.16-1.46)**  **1.20 (1.07-1.35)**  1.16 (0.96-1.39)  **-**  **1.22 (1.09-1.37)**  **1.31 (1.16-1.47)**  **1.45 (1.30-1.61)** | **<.001**  **<.001**  **.003**  .13  **<.001**  **<.001**  **<.001** | **1.19 (1.06-1.33)**  **1.20 (1.06-1.36)**  1.06 (0.94-1.21)  **1.41 (1.16-1.73)**  **-**  1.11 (0.98-1.25)  1.12 (0.98-1.28)  **1.25 (1.11-1.42)** | **.003**  **.005**  .35  **<.001**  .10  .10  **<.001** |
| **Occupational exposure (individual agents)** | | | | | | |
| Animals  Fish/shellfish  Flour  Foods  Plants-related dusts  House dust mites  Storage mites  Plant mites  Enzymes  Latex  Textiles  Moulds  Endotoxin  Drugs  High-level chemical disinfectants  Aliphatic amines  Isocyanates  Acrylates  Epoxy resins  Persulfates/henna  Wood  Metal  Metal working fluids  Herbicides  Insecticides  Fungicides  Indoor cleaning  Bleach  Organic solvents  Exhaust fumes | 31 (2.0)  12 (0.8)  <10 (<0.6)  <10 (<0.6)  13 (0.8)  84 (5.4)  30 (1.9)  13 (0.8)  <10 (<0.6)  312 (19.9)  <10 (<0.6)  35 (2.2)  38 (2.4)  17 (1.1)  293 (18.7)  33 (2.1)  <10 (<0.6)  14 (0.9)  10 (0.6)  25 (1.6)  11 (0.7)  34 (2.2)  <10 (<0.6)  13 (0.8)  48 (3.1)  33 (2.1)  397 (25.4)  251 (16.0)  179 (11.4)  85 (5.4) | 988 (2.1)  368 (0.8)  268 (0.6)  284 (0.6)  625 (1.3)  1894 (3.9)  1006 (2.1)  625 (1.3)  268 (0.6)  6899 (14.3)  194 (0.4)  1389 (2.9)  1443 (3.0)  449 (0.9)  6874 (14.3)  799 (1.7)  348 (0.7)  563 (1.2)  568 (1.2)  458 (1.0)  607 (1.3)  1677 (3.5)  226 (0.5)  659 (1.4)  1612 (3.4)  1251 (2.6)  8942 (18.6)  5669 (11.8)  5319 (11.1)  3355 (7.0) | **-**  **-**  **-**  **-**  **-**  **-**  **-**  **-**  **-**  **1.49 (1.31-1.69)**  **-**  **-**  **-**  **-**  **1.38 (1.21-1.57)**  **-**  **-**  **-**  **-**  **-**  **-**  **-**  **-**  **-**  **-**  **-**  **1.49 (1.33-1.67)**  **1.43 (1.25-1.64)**  1.04 (0.89-1.22)  **-** | **<.001**  **<.001**  **<.001**  **<.001**  .64 | **-**  **-**  **-**  **-**  **-**  **-**  **-**  **-**  **-**  **1.18 (1.03-1.36)**  **-**  **-**  **-**  **-**  1.11 (0.96-1.28)  **-**  **-**  **-**  **-**  **-**  **-**  **-**  **-**  **-**  **-**  **-**  **1.31 (1.15-1.50)**  **1.20 (1.03-1.40)**  1.04 (0.88-1.24)  **-** | **.02**  .17  **<.001**  **.02**  .65 |
| Number of exposures to individual agents, mean ±SD  Missing | 1.3 ±1.9  <10 | 1.1 ±1.9   327 | **1.04 (1.02-1.07)** | **.001** | **1.04 (1.01-1.07)** | **.006** |

*Note: P*-values ≤0.05 are shown in bold.

Data on occupational exposure was derived from linkage with the Occupational asthma-specific job exposure matrix by Kennedy et al. and Le Moual et al., according to most recently reported International Standard Classification of Occupations version 2008 (ISCO-08) coded occupation.^6,15,17^ Binary logistic regression analyses were only presented when at least 100 cases and controls were available.

**^†^**Adjusted for age, sex and atopic dermatitis.

Abbreviations: *CI,* confidence interval; *HE*, hand eczema; *HMW,* high molecular weight; *LMW,* low molecular weight; *n*, number; *OR,* Odds ratio.

**Table S3.** Associations between different levels of occupational exposure and moderate-to-very severe hand eczema at worst in the past year vs no hand eczema in lifetime (N=49 995).

|  | **Moderate-to-very severe HE in the past year, n (%) (n=1572)** | **No HE ever, n (%) (n=48423)** | **Crude OR (95% CI)** | ***P-*value** | **Adjusted OR^†^ (95% CI)** | ***P-*value** |
| --- | --- | --- | --- | --- | --- | --- |
| **Occupational exposure (groups)** | | | | | | |
| Irritants  No  Medium  High  HMW sensitizers  No  Medium  High  LMW sensitizers  No  Medium  High  Mites  No  Medium  High  Microbial exposure  No  Medium  High  Highly reactive chemicals  No  Medium  High  Biocides  No  Medium  High  Disinfectants and cleaning products  No  Medium  High  Missing | 921 (58.8)  402 (25.7)  242 (15.5)  1156 (73.9)  241 (15.4)  168 (10.7)  1183 (75.6)  309 (19.7)  73 (4.7)  1441 (92.1)  86 (5.5)  38 (2.4)  1516 (96.9)  24 (1.5)  25 (1.6)  1126 (71.9)  261 (16.7)  178 (11.4)  1208 (77.2)  207 (13.2)  150 (9.6)  1087 (69.5)  311 (19.9)  167 (10.7) | 30493 (63.4)  10758 (22.4)  6845 (14.2)  37823 (78.6)  5870 (12.2)  4403 (9.2)  37881 (78.8)  80000 (16.6)  2215 (4.6)  44765 (93.1)  2200 (4.6)  1131 (2.4)  46192 (96.0)  1031 (2.1)  873 (1.8)  36458 (75.8)  7410 (15.4)  4228 (8.8)  39225 (81.6)  5259 (10.9)  3612 (7.5)  36879 (76.7)  7391 (15.4)  3826 (8.0) | 1.00 (ref)  **1.24 (1.10-1.39)**  **1.17 (1.01-1.35)**  1.00 (ref)  **1.34 (1.17-1.55)**  **1.25 (1.06-1.47)**  **-**  **-**  **-**  **-**  **-**  **-**  1.00 (ref)  1.14 (0.99-1.31)  **1.36 (1.16-1.60)**  1.00 (ref)  **1.28 (1.10-1.49)**  **1.35 (1.13-1.60)**  1.00 (ref)  **1.43 (1.26-1.62)**  **1.48 (1.25-1.75)** | **<.001**  **.03**  **<.001**  **.008**  .06  **<.001**  **.001**  **<.001**  **<.001**  **<.001** | 1.00 (ref)  **1.17 (1.02-1.33)**  **1.22 (1.05-1.43)**  1.00 (ref)  **1.22 (1.05-1.43)**  1.17 (0.97-1.39)  **-**  **-**  **-**  **-**  **-**  **-**  1.00 (ref)  1.03 (0.89-1.19)  **1.26 (1.05-1.50)**  1.00 (ref)  1.02 (0.87-1.21)  **1.28 (1.06-1.55)**  1.00 (ref)  **1.22 (1.06-1.41)**  **1.32 (1.10-1.59)** | **.02**  **.01**  **.01**  .10  .72  **.01**  .79  **.01**  **.007**  **.003** |
| **Occupational exposure (individual agents)** | | | | | | |
| Animals  No  Medium  High  Fish/shellfish  No  Medium  High  Flour  No  Medium  High  Foods  No  Medium  High  Plants-related dusts  No  Medium  High  House dust mites  No  Medium  High  Storage mites  No  Medium  High  Plant mites  No  Medium  High  Enzymes  No  Medium  High  Latex  No  Medium  High  Textiles  No  Medium  High  Moulds  No  Medium  High  Endotoxin  No  Medium  High  Drugs  No  Medium  High  High-level chemical disinfectants  No  Medium  High  Aliphatic amines  No  Medium  High  Isocyanates  No  Medium  High  Acrylates  No  Medium  High  Epoxy resins  No  Medium  High  Persulfates/henna  No  Medium  High  Wood  No  Medium  High  Metal  No  Medium  High  Metal working fluids  No  Medium  High  Herbicides  No  Medium  High  Insecticides  No  Medium  High  Fungicides  No  Medium  High  Indoor cleaning  No  Medium  High  Bleach  No  Medium  High  Organic solvents  No  Medium  High  Exhaust fumes  No  Medium  High  Missing | 1534 (98.0)  <10 (<0.6)  23 (1.5)  1553 (99.2)  12 (0.8)  0 (0.0)  1558 (99.6)  <10 (<0.6)  <10 (<0.6)  1558 (99.6)  <10 (<0.6)  0 (0.0)  1552 (99.2)  <10 (<0.6)  10 (0.6)  1481 (94.6)  56 (3.6)  28 (1.8)  1535 (98.1)  30 (1.9)  0 (0.0)  1552 (99.2)  <10 (<0.6)  10 (0.6)  1558 (99.6)  <10 (<0.6)  0 (0.0)  1253 (80.1)  218 (13.9)  94 (6.0)  1560 (99.7)  <10 (<0.6)  0 (0.0)  1530 (97.8)  34 (2.2)  <10 (<0.6)  1527 (97.6)  15 (1.0)  23 (1.5)  1548 (98.9)  <10 (<0.6)  14 (0.9)  1272 (81.3)  270 (17.3)  23 (1.5)  1532 (97.9)  31 (2.0)  <10 (<0.6)  1559 (99.6)  <10 (<0.6)  <10 (<0.6)  1551 (99.1)  14 (0.9)  0 (0.0)  1555 (99.4)  <10 (<0.6)  <10 (<0.6)  1540 (98.4)  0 (0.00  25 (1.6)  1554 (99.3)  10 (0.6)  <10 (<0.6)  1531 (97.8)  33 (2.1)  <10 (<0.6)  1560 (99.7)  <10 (<0.6)  <10 (<0.6)  1552 (99.2)  <10 (<0.6)  <10 (<0.6)  1517 (96.9)  35 (2.2)  13 (0.8)  1532 (97.9)  22 (1.4)  11 (0.7)  1168 (74.6)  253 (16.2)  144 (9.2)  1314 (84.0)  137 (8.8)  114 (7.3)  1386 (88.6)  146 (9.3)  33 (2.1)  1480 (94.6)  64 (4.1)  21 (1.3) | 47108 (97.9)  313 (0.7)  675 (1.4)  47728 (99.2)  358 (0.7)  10 (0.0)  47828 (99.4)  195 (0.4)  73 (0.2)  47812 (99.4)  284 (0.6)  0 (0.0)  47471 (98.7)  212 (0.4)  413 (0.9)  46202 (96.1)  1176 (2.4)  718 (1.5)  47090 (97.9)  1006 (2.1)  0 (0.0)  47471 (98.7)  212 (0.4)  413 (0.9)  47828 (99.4)  268 (0.6)  0 (0.0)  41197 (85.7)  4733 (9.8)  2166 (4.5)  47902 (99.6)  181 (0.4)  13 (0.0)  46707 (97.1)  1330 (2.8)  59 (0.1)  46653 (97.0)  693 (1.4)  750 (1.6)  47647 (99.1)  95 (0.2)  354 (0.7)  41222 (85.7)  6208 (12.9)  666 (1.4)  47297 (98.3)  706 (1.5)  93 (0.2)  47748 (99.3)  320 (0.7)  28 (0.1)  47533 (98.8)  563 (1.2)  0 (0.0)  47528 (98.8)  150 (0.3)  418 (0.9)  47638 (99.0)  0 (0.0)  458 (1.0)  47489 (98.7)  530 (1.1)  77 (0.2)  46419 (96.5)  1528 (3.2)  149 (0.3)  47870 (99.5)  116 (0.2)  110 (0.2)  47437 (98.6)  414 (0.9)  245 (0.5)  46484 (96.6)  1020 (2.1)  592 (1.2)  46845 (97.4)  752 (1.6)  499 (1.0)  39154 (81.4)  5782 (12.0)  3160 (6.6)  42427 (88.2)  3319 (6.9)  2350 (4.9)  42777 (88.9)  4590 (9.5)  729 (1.5)  44741 (93.0)  2240 (4.7)  1115 (2.3) | **-**  **-**  **-**  **-**  **-**  **-**  **-**  **-**  **-**  **-**  **-**  **-**  **-**  **-**  **-**  **-**  **-**  **-**  **-**  **-**  **-**  **-**  **-**  **-**  **-**  **-**  **-**  **-**  **-**  **-**  **-**  **-**  **-**  **-**  **-**  **-**  **-**  **-**  **-**  **-**  **-**  **-**  **-**  **-**  **-**  **-**  **-**  **-**  **-**  **-**  **-**  **-**  1.00 (ref)  **1.47 (1.28-1.69)**  **1.53 (1.28-1.82)**  1.00 (ref)  **1.33 (1.11-1.60)**  **1.57 (1.29-1.91)**  **-**  **-**  **-**  **-** | **<.001**  **<.001**  **.002**  **<.001** | **-**  **-**  **-**  **-**  **-**  **-**  **-**  **-**  **-**  **-**  **-**  **-**  **-**  **-**  **-**  **-**  **-**  **-**  **-**  **-**  **-**  **-**  **-**  **-**  **-**  **-**  **-**  **-**  **-**  **-**  **-**  **-**  **-**  **-**  **-**  **-**  **-**  **-**  **-**  **-**  **-**  **-**  **-**  **-**  **-**  **-**  **-**  **-**  **-**  **-**  **-**  **-**  1.00 (ref)  **1.33 (1.14-1.55)**  **1.29 (1.05-1.57)**  1.00 (ref)  1.16 (0.95-1.41)  **1.27 (1.02-1.58)**  **-**  **-**  **-**  **-** | **<.001**  **.01**  .15  **.04** |

*Note: P*-values ≤0.05 are shown in bold.

Data on occupational exposure was derived from linkage with the Occupational asthma-specific job exposure matrix by Kennedy et al. and Le Moual et al., according to most recently reported International Standard Classification of Occupations version 2008 (ISCO-08) coded occupation.^6,15,17^ Binary logistic regression analyses were only presented when at least 100 cases and controls were available.

**^†^**Adjusted for age, sex and atopic dermatitis.

Abbreviations: *CI,* confidence interval; *HE*, hand eczema; *HMW,* high molecular weight; *LMW,* low molecular weight; *n*, number; *OR,* Odds ratio.

**References**

1	Susitaival P, Flyvholm MA, Meding B *et al.* Nordic Occupational Skin Questionnaire (NOSQ-2002): a new tool for surveying occupational skin diseases and exposure. *Contact Dermatitis* 2003; **49**: 70-6.

2	Meding B, Barregård L. Validity of self-reports of hand eczema. *Contact Dermatitis* 2001; **45**: 99-103.

3	Smit HA, Coenraads PJ, Lavrijsen AP, Nater JP. Evaluation of a self-administered questionnaire on hand dermatitis. *Contact Dermatitis* 1992; **26**: 11-6.

4	Coenraads PJ, Van Der Walle H, Thestrup-Pedersen K *et al.* Construction and validation of a photographic guide for assessing severity of chronic hand dermatitis. *Br J Dermatol* 2005; **152**: 296-301.

5	Hald M, Veien NK, Laurberg B, Johansen JD. Severity of hand eczema assessed by patients and dermatologist using a photographic guide. *Contact Dermatitis* 2007; **156**: 77-80.

6	International Labour Organization. International Standard Classification of Occupations (ISCO) Available at: <https://ilostat.ilo.org/resources/concepts-and-definitions/classification-occupation/>. Accessed: January 16.

7	Barbarot S, Auziere S, Gadkari A *et al.* Epidemiology of atopic dermatitis in adults: Results from an international survey. *Allergy* 2018; **73**: 1284-93.

8	Voorberg AN, Loman L, Schuttelaar MLA. Prevalence and Severity of Hand Eczema in the Dutch General Population: A Cross-sectional, Questionnaire Study within the Lifelines Cohort Study. *Acta Derm Venereol* 2022; **102**: adv00626.

9	Brands MJ, Loman L, Lund TT *et al.* The association between wet work and hand eczema in the Dutch general population: Application of a job exposure matrix to the lifelines cohort study. *Contact Dermatitis* 2024; **92(1)**: 31-40.

10	Brands MJ, Loman L, Roelen CAM *et al.* Hand eczema-related presenteeism and sickness absence: A cross-sectional population-based study. *Contact Dermatitis* 2024; **90**: 372-7.

11	Brands MJ, Loman L, Schuttelaar MLA. Exposure and work‐related factors in subjects with hand eczema: Data from a cross‐sectional questionnaire within the Lifelines Cohort Study. *Contact Dermatitis* 2022; **86**: 493-506.

12	Loman L, Brands MJ, Politiek K, Schuttelaar MLA. Health Literacy and Associated Factors in Subjects with Hand Eczema: A Dutch Population-based Study. *Acta Derm Venereol* 2024; **104**: adv40079.

13	Loman L, Politiek K, Schuttelaar MLA. Smoking and obesity are associated with chronic hand eczema and severity of hand eczema: Data from the Dutch general population. *Contact Dermatitis* 2022; **87**: 103-6.

14	Loman L, Schuttelaar MLA. Hand eczema and lifestyle factors in the Dutch general population: Evidence for smoking, chronic stress, and obesity. *Contact Dermatitis* 2022; **86**: 80-8.

15	Moual NL, Zock JP, Dumas O *et al.* Update of an occupational asthma-specific job exposure matrix to assess exposure to 30 specific agents. *Occupational and Environmental Medicine* 2018; **75**: 507-14.

16	Sit G, Varraso R, Fezeu LK *et al.* Occupational Exposures to Irritants and Sensitizers, Asthma and Asthma Control in the Nutrinet-Santé Cohort. *J Allergy Clin Immunol Pract* 2022; **10**: 3220-7.e7.

17	Kennedy SM, Le Moual N, Choudat D, Kauffmann F. Development of an asthma specific job exposure matrix and its application in the epidemiological study of genetics and environment in asthma (EGEA). *Occup Environ Med* 2000; **57**: 635-41.
